# Ethnoveterinary medicines used for ruminants in British Columbia, Canada

**DOI:** 10.1186/1746-4269-3-11

**Published:** 2007-02-26

**Authors:** Cheryl Lans, Nancy Turner, Tonya Khan, Gerhard Brauer, Willi Boepple

**Affiliations:** 1 BCICS, University of Victoria, British Columbia, V8W 2Y2, Canada; 2 School of Environmental Studies, University of Victoria, British Columbia, V8W 3P5, Canada; 3 DVM, Vancouver, British Columbia, Canada; 4 School of Health Information Science, University of Victoria, British Columbia, V8W 3P5, Canada; 5 Canadian Liaison National Saanen Breeders. 499 Millstream Lake Rd. Victoria, B.C., Canada, V9E 1K2

## Abstract

**Background:**

The use of medicinal plants is an option for livestock farmers who are not allowed to use allopathic drugs under certified organic programs or cannot afford to use allopathic drugs for minor health problems of livestock.

**Methods:**

In 2003 we conducted semi-structured interviews with 60 participants obtained using a purposive sample. Medicinal plants are used to treat a range of conditions. A draft manual prepared from the data was then evaluated by participants at a participatory workshop.

**Results:**

There are 128 plants used for ruminant health and diets, representing several plant families. The following plants are used for abscesses: *Berberis aquifolium*/*Mahonia aquifolium Echinacea purpurea*, *Symphytum officinale*, *Bovista pila*, *Bovista plumbea*, *Achillea millefolium *and *Usnea longissima*. *Curcuma longa *L., *Salix scouleriana *and *Salix lucida *are used for caprine arthritis and caprine arthritis encephalitis.*Euphrasia officinalis *and *Matricaria chamomilla *are used for eye problems.

Wounds and injuries are treated with *Bovista *spp., *Usnea longissima*, *Calendula officinalis*, *Arnica *sp., *Malva *sp., *Prunella vulgaris*, *Echinacea purpurea*, *Berberis aquifolium*/*Mahonia aquifolium*, *Achillea millefolium*, *Capsella bursa*-*pastoris*, *Hypericum perforatum*, *Lavandula officinalis*, *Symphytum officinale *and *Curcuma longa*.

*Syzygium aromaticum *and *Pseudotsuga menziesii *are used for coccidiosis. The following plants are used for diarrhea and scours: *Plantago major*, *Calendula officinalis*, *Urtica dioica*, *Symphytum officinale*, *Pinus ponderosa*, *Potentilla pacifica*, *Althaea officinalis*, *Anethum graveolens*, *Salix alba *and *Ulmus fulva*.

Mastitis is treated with *Achillea millefolium*, *Arctium lappa*, *Salix alba*, *Teucrium scorodonia *and *Galium aparine*. *Anethum graveolens *and *Rubus *sp., are given for increased milk production.*Taraxacum officinale*, *Zea mays*, and *Symphytum officinale *are used for udder edema. Ketosis is treated with *Gaultheria shallon*, *Vaccinium *sp., and *Symphytum officinale*. *Hedera helix *and *Alchemilla vulgaris *are fed for retained placenta.

**Conclusion:**

Some of the plants showing high levels of validity were *Hedera helix *for retained placenta and *Euphrasia officinalis *for eye problems. Plants with high validity for wounds and injuries included *Hypericum perforatum*, *Malva parviflora *and *Prunella vulgaris*. Treatments with high validity against endoparasites included those with *Juniperus communis *and *Pinus ponderosa*. Anxiety and pain are well treated with *Melissa officinalis *and *Nepeta caesarea*.

## Background

Our research co-operatively documented and validated (in a non-experimental way) the ethnoveterinary medicines used by livestock farmers in British Columbia. As scientists we evaluated technology already developed by farmers or community members. Ethnoveterinary medicine is the scientific term for traditional animal health care. Research into ethnoveterinary medicine is often undertaken as part of a community-based approach that serves to improve animal health and provide basic veterinary services in rural areas. The research area of British Columbia had 383 organic farms in 2004, a decline of 1.5% since 2001, on approximately 25,000 acres [10,000 ha]. This represents 1.9% of all farms. There are an additional 77 farms in transition to certified organic production [1]. Only 1.5% of the population of British Columbia lives on a farm [2].

The average wage for farmers working full time in agriculture in the Capital Region of Vancouver Island was $14,000; however 53% of all farms have receipts of less than $5000. It was reported that 7,460 farmers in British Columbia with annual sales of over $10,000 have a low net farm income. The return to assets on these farms ranges from -1% for farmers with sales of $19,000 to $25,000 to 5.2% from farms with sales of over $250,000. Only 13% of farmers report receipts of over $25,000 [2]. In 2003 there were 420 certified organic farmers 51% of which had less than $10,000 in gross sales [1]. Twenty percent of these organic farmers had over $50,000 in gross sales [1]. These figures are important because sustainable agriculture has been defined (by the Federal-Provincial Agriculture Committee on Environmental Sustainability) as that which is economically viable for the present generation of farmers and environmentally sustainable for the future generation [3,4].

## Materials and methods

The research tested the potential of participatory workshops as a dissemination activity or new way of transferring knowledge in ethnoveterinary medicine. The International Institute of Rural Reconstruction (IIRR) developed the workshop method and it is said to have two major advantages: it reduces the total amount of time needed to develop information materials (a user-friendly manual) and it profits from the expertise and resources of a wide range of participants and their organizations. The remedies chosen for inclusion in the manual are those that can be recommended for use by the general public and farmers to alleviate minor diseases and problems. The produced manual can provide a sustainable long-term solution to animal health problems. The workshop method allows participants to pool resources, abilities and information thus multiplying the likelihood of obtaining useful solutions and minimizing the risk of failure.

Ethnoveterinary data for British Columbia was collected over a six-month period in 2003. All available literature about livestock farmers and the secondary literature on ethnomedicinal plants, folk medicine and related fields in British Columbia was reviewed prior to and during the research [5-12]. The research area in British Columbia consisted of the Lower Mainland, the Thompson/Okanagan region and south Vancouver Island.

A purposive sample of livestock farmers was created to target key informants with the knowledge sought. The sample size was 60. The sample was obtained from membership lists of organic farmers, other specialists in alternative medicine and holistic veterinarians.

Seven of the participants with ruminants had goats and a few had cows; these provided the majority of the information recorded in this paper. Other information came from holistic practitioners, herbalists, holistic veterinarians and participants with horses and pets.

Two visits were made to each farm or respondent. All of the interviews at the initial stage were open-ended and unstructured. A draft outline of the respondents' ethnoveterinary remedies was delivered and discussed at the second visit in order to confirm the information provided at the first interview. Medicinal plant voucher specimens were collected where possible and were identified and deposited in the University of Victoria Herbarium.

The plant-based remedies were evaluated for safety and efficacy with a non-experimental method, prior to including them in the draft outline. Published sources such as journal articles and books and databases on pharmacology and ethnomedicine available on the Internet were searched to identify the plants' chemical compounds and clinically tested physiological effects. This data was incorporated with data on the reported folk uses, and their preparation and administration in North America and Europe. For each species or genus the ethnomedicinal uses in other countries are given; followed by a summary of chemical constituents, in addition to active compounds if known. This type of ethnopharmacological review and evaluation is based on previous work and the use of these methods in the same research study has been published [4]. The non-experimental validation of the plants is presented in the discussion section of the paper.

### Validation workshop

Ten participants with experience in traditional human and ethnoveterinary medicine took part in a participatory five-day-long workshop at the University of Victoria (BC), in October, 2003. In the workshop the facilitator asked participants very specific questions in a supportive environment about the medicinal plants used. Each animal/livestock species was covered in a morning or afternoon session, other than the core group, different participants came to different sessions [4]. At the ruminant session the four participants (herbalists and ruminant owners) introduced themselves and their work and were instructed on the participatory workshop method. The participants discussed the previously produced ruminant section of the data. There were two editorial assistants/facilitators in attendance. After the discussions, the ruminant section was edited.

### Non-experimental validation of ethnoveterinary remedies

The researcher and the ethnoveterinary consultant completed the non-experimental validation of the remedies in advance of the workshop. A low-cost, non-experimental method was used to evaluate the potential efficacy of the ethnoveterinary remedies [4]. This method consisted of:

• obtaining an accurate botanical identification of the herbal remedies reported;

• searching the pharmaceutical/pharmacological literature for the plant's identified chemical constituents in order to determine the known physiological effects of either the crude plant drug, related species, or isolated chemical compounds that the plant is known to contain. This information was then used to assess whether the plant use is based on empirically verifiable principles.

Supporting ethnobotanical data and pharmacological information was matched with the recorded folk use of the plant species [5-12], to determine degrees of confidence about its effectiveness. Four levels of confidence were established:

1. Minimal level: If no information supports the use it indicates that the plant may be inactive.

2. Low level: A plant (or closely related species of the same genus), which is used in distinct areas in the treatment of similar illnesses (humans or preferably animals), attains the lowest level of validity, if no further phytochemical or pharmacological information validates the popular use. Use in other areas increases the likelihood that the plant is efficacious.

3. Mid level: If in addition to the ethnobotanical data, available phytochemical or pharmacological information is consistent with the use, this indicates a higher level of confidence that the plant may exert a physiological action on the patient.

4. High level: If both ethnobotanical and pharmacological data are consistent with the folk use of the plant, its use is classed in the highest level of validity and is considered efficacious.

## Results

One hundred and twenty-eight plants are used in total. There are 78 plants used for health and diet in ruminants that represent several plant families (Table 1). Fifty-four plants from many plant families are used as food (Table 2). Eleven plants are considered poisonous (Table 3). Eleven plants are used specifically during pregnancy (Table 4). All of the results were discussed at the workshop and included in a practical manual on ethnoveterinary medicine (EVM) in B.C. that was given to each participant. The results are outlined by category below.

**Table 1:** Ethnoveterinary medicines used for ruminants in British Columbia

Scientific name, (botanical family) Voucher specimen number	Local name	Part(s) used	Ethnoveterinary use
*Acer macrophyllum *Pursh (Aceraceae) JB043	Big leaf maple	leaves	bedding
*Achillea millefolium *L. (Asteraceae) JS 041	yarrow	Aerial parts	Mastitis, wounds, sternal abscess
*Achlys triphylla *(Smith) DC. (Berberidaceae) JS018	Vanilla leaf	leaves	flies
*Alchemilla vulgaris *L. (Rosaceae) JS011	Lady's mantle	aerial parts	Retained placenta
*Allium cepa *L. (Alliaceae) not collected	onion	Skins	endoparasites
*Allium sativum *L. (Amaryllidaceae) not collected	garlic	minced cloves	Endoparasites, respiratory tonic
*Althaea officinalis *L. (Malvaceae) not collected	marshmallow	Aerial parts	Diarrhea, scours
*Anethum graveolens *L. (Apiaceae) JS010	Dill	seed	Diarrhea, scours, milk production
*Apium graveolens *L. (Apiaceae) not collected	celery	Aerial parts	endoparasites
*Arctium lappa *L. (Asteraceae) JB32	Burdock	root	mastitis
*Arnica *sp. (Asteraceae) JB92	Wild arnica	Leaves or flowers	wounds
*Artemisia *sp. (Compositae) JS105	wormwood	leaves	endoparasites
*Artemisia vulgaris *L. (Asteraceae) JS016	mugwort	plant	Zinc deficiency
*Azadirachta indica *A. Juss. purchased product	neem	powder	lice
*Berberis aquifolium *Pursh. *Mahonia aquifolium *(Berberidaceae) JB6	Oregon grape	roots	wounds
*Blechnum spicant *(L.) Roth (Polypodiaceae) not collected	Deer fern	Aerial parts	Magnesium imbalance
*Bovista pila *Berk. & M. A. Curtis, *Bovista plumbea *Pers. (Lycoperdaceae) JB1	puffball	Spore mass	Disbudding, cuts, wounds, sternal abscess
*Calendula officinalis *L. (Asteraceae) JB84	calendula	Flower oil	Cuts, scratches, diarrhea, sore stomachs
*Capsella bursa*-*pastoris *(L.) Medic. (Brassicaceae) not collected	shepherd's purse	leaves	wounds
*Cinnamomum zeylanicum *Blume (Lauraceae) purchased product	cinnamon	Inner bark	Diarrhea, scours
*Curcuma longa *L. (Zingiberaceae) purchased product	turmeric	rhizome	Caprine arthritis encephalitis palliative, proud flesh
*Cymbopogon nardus *(L.) Rendle (Poaceae) purchased product	citronella	oil	flies
*Daucus carota *L. (Apiaceae) not collected	carrots	Roots & tops	endoparasites
*Echinacea purpurea *(L.) Moench (Asteraceae) JBCL 07	Echinacea	root	abscess, Pre-show protection, wounds, respiratory tonic
*Epilobium augustifolium *L. (Onagraceae) not collected	fireweed	Fresh or dry plant	Appetite stimulant
*Eugenia caryophyllata *Thunb (Myrtaceae) purchased product	cloves	Essential oil	flies
*Euphrasia officinalis *agg. (Scrophulariaceae) purchased product	eyebright	Aerial parts	Eye problems
*Galium aparine *L. (Rubiaceae) JB3	cleavers	Aerial parts	mastitis
*Gaultheria shallon *Pursh. (Ericaceae) JS014	salal	Aerial parts	Rumen tonic, ketosis
*Hedera helix *L. (Araliaceae) not collected	English ivy	leaves	Retained placenta
*Helianthus annuus *L. (Asteraceae) purchased product	sunflower	seeds	endoparasites
*Hypericum perforatum *L. (Hypericaceae) JS027	St. John's Wort	Infused oil of flowers	Proud flesh, wounds
*Juniperus communis *L. (Cupressaceae)	juniper	branches	Endoparasites, liver fluke
*Lavandula officinalis *L. (Labiatae)	lavender	Essential oil	Flies, proud flesh
*Mahonia nervosa *(Pursh) Nutt (Berberidaceae) JS104	Oregon grape	Root decoction	Abscess, Respiratory tonic
*Malva sylvestris *(Malvaceae) JS002	mallow	plant	wounds
*Matricaria chamomilla *L. (Compositae) JB43	chamomile	flower	eye problems
*Medicago sativa *L. (Leguminosae) purchased product	alfalfa	Pellets from high-selenium regions, aerial parts	Selenium deficiency, nutrition after calving
*Melaleuca alternifolia *L. (Myrtaceae) purchased product	Tea tree oil	drops	endoparasites
*Mentha piperita *L. (Lamiaceae) JS024	peppermint	Essential oil	flies
*Mentha pulegium *L. (Lamiaceae)	pennyroyal	Aerial parts	Flies, lice
*Nepeta cataria *L. (Lamiaceae) not collected	catnip	Aerial parts	Pain killer
*Pastinaca sativa *L. (Apiaceae) not collected	parsnip	tops	endoparasites
*Petroselinum crispum *L. (Apiaceae) not collected	parsley	Aerial parts	endoparasites
*Pinus ponderosa *Douglas ex Lawson (Pinaceae) JB98	long needle yellow pine	branches	Diarrhea grey pasty young animals, endoparasites
*Plantago lanceolata *L. (Plantaginaceae) JS042	plantain	leaves	diarrhea
*Polystichum munitum *(Kaulf.) Presl. (Polypodiaceae) JS047	Sword fern	Aerial parts	Stimulate digestion
*Portulaca oleracea *L. Portulacaceae not collected	purslane	shoot	Zinc deficiency
*Potentilla recta*L. (Rosaceae) JB93	cinquefoil	Aerial parts	Appetite stimulant, diarrhea, scours
*Prunella vulgaris *L. (Lamiaceae) JS111	self heal	plant	wounds
*Pseudotsuga menziesii *(Mirbel) Franco (Pinaceae) JS049	Douglas fir	Top branches	Appetite stimulant, coccidiosis, endoparasites
*Quercus alba *L. (Fagaceae) not collected	White oak	shoot	Zinc deficiency
*Rubus *sp. (Rosaceae) not collected	thornless raspberry	leaves	Milk production
*Rubus ursinus *L. JS115 and *Rubus laciniatus *Willd. (Rosaceae)	blackberry	leaves	Unknown illness
*Ruta graveolens *L. (Rutaceae) not collected	rue	leaves	flies
*Salix alba *L. var. sericea Gaudin (Salicaceae) not collected	White willow	bark	Diarrhea, scours, mastitis, pain, unknown illness
*Salix scouleriana *Barratt ex Hook, *Salix lucida *Muhl. ssp. *lasiandra *(Benth.) E. Murr. (Salicaceae) JS101	Scoulers willow, Pacific willow	Branches	Caprine arthritis encephalitis palliative
*Salvia officinalis *L. (Lamiaceae) JS035	Garden sage	leaves	Drying off
*Senna *sp. (Fabaceae) purchased product	senna	pod	Diarrhea, scours
*Symphoricarpos albus*var. *laevigatus *(Caprifoliaceae) JS103	snowberry	branches	endoparasites
*Symphytum officinale *L. (Boraginaceae) JBCL 08	comfrey	Leaves fresh/dry	Abscess, wounds, diarrhea, flystrike, proudflesh
*Symphytum officinale *L. (Boraginaceae) JBCL 08	comfrey	Aerial parts	Increase butterfat; laxative, ketosis, udder edema
*Syzygium aromaticum *(L.) Merr. & Perry. (Myrtaceae) purchased product	cloves	bud	coccidia
*Taraxacum officinale *(L.) Weber (Asteraceae) JB96	dandelions	leaves	Udder edema
*Teucrium scorodonia *L. (Labiatae) not collected	woodsage	Tincture	mastitis
*Thuja plicata *Donn ex D. Don (Cupressaceae) JBR 21	cedar	Bark shavings	lice
*Thuja plicata *Donn ex D. Don (Cupressaceae) JS036	Red cedar	branches	Copper deficiency, endoparasites
*Ulmus fulva *Michx. (Ulmaceae) purchased product	Slippery elm	Bark powder	Blood in stool
*Ulmus fulva *Michx. (Ulmaceae) purchased product	Slippery elm	Inner bark	Diarrhea, scours
*Urtica dioica *L. (Urticaceae) JS023	nettle	Ground seeds	endoparasites
*Urtica dioica *L. (Urticaceae) JS023	nettles	Aerial parts	Pre-show protection, zinc deficiency, diarrhea
*Usnea longissima *Ach. (Parmeliaceae) JB2a	usnea	Aerial parts	Dehorning adults, foot rot, sternal abscess
*Vaccinium parvifolium *Sm. (Ericaceae) JS045	huckleberry	plant	ketosis
*Valeriana officinalis *L. (Valerianaceae) JS008	valerian	roots	Pain killer
*Verbascum thapsus *L. (Scrophulariaceae) JS118	mullein	flower	Respiratory tonic
*Vitis *sp. (Vitaceae) not collected	grape	leaves	Unknown illness
*Zea mays *L. (Poaceae) not collected	cornsilk	Silk (style, stigma)	Udder edema
*Zingiber officinale *Roscoe (Zingiberaceae) purchased product	ginger	rhizome	Diarrhea, scours

**Table 2:** Plants used as food for ruminants in British Columbia

Scientific name	Local name	Part(s) used	Additional Use
*Fucus vesiculosus *L. (Fucaceae; Brown Algae) JBCL 11	kelp	Plant	Healthy fleece
*Fucus vesiculosus *L. (Fucaceae; Brown Algae) JBCL 11	bladderwrack	Plant	Iodine, trace minerals
*Abies grandis *(Douglas ex D. Don) Lindley not collected	grand fir	Branches	maintain body heat
*Acer macrophyllum *Pursh (Aceraceae) JB 043	big-leaf maple	inner bark	--
*Achillea millefolium *L. (Asteraceae) JS041	yarrow	aerial parts	--
*Achillea millefolium *L. (Asteraceae) JS041	yarrow	aerial parts	--
*Achlys triphylla *(Smith) de Candolle (Berberidaceae) JS018	vanilla leaf	aerial parts	--
*Adenocaulon bicolour *Hook. (Asteraceae) not collected	silver-green/pathfinder	aerial parts	--
*Alnus rubra *Bong. (Betulaceae) JB 108	red alder	aerial parts	maintain body heat
*Anaphalis margaritacea *(L.) Benth. (Asteraceae) JS034	pearly everlasting	aerial parts	--
*Arbutus menziesii *Pursh (Ericaceae) JS013	Arbutus	fresh and dried leaves	maintain body heat
*Arctium lappa *L. (Asteraceae) JB32	Burdock	aerial parts	--
*Artemisia dracunculus *L. (Asteraceae) JS025	Tarragon	aerial parts	--
*Berberis aquifolium/Mahonia aquifolium *(Berberidaceae) JB79	Oregon grape	aerial parts	--
*Chenopodium album *L. (Chenopodiaceae) JBR 94	lamb's quarters	aerial parts	--
*Cichorium intybus *L. (Asteraceae) not collected	Chicory	aerial parts	--
*Cirsium arvense *(L.) Scop. (Asteraceae) JS030	Canada thistle	aerial parts	Vitamin A
*Claytonia perfoliata *Donn ex Willd. ssp. *perfoliata *(Portulacaceae) JB20	miners lettuce	aerial parts	--
*Cornus sericea *(Cornaceae) not collected	red osier dogwood	aerial parts	Winter feed
*Crepis capillaris *(L.) Wallr. (Asteraceae) JS106	Crepis/Hawk's beard	aerial parts	--
*Cucurbita pepo *L. (Cucurbitaceae) not collected	pumpkin	Fruit	Vitamin A
*Cystisus scoparius *L (Leguminosae)	broom	aerial parts	cardiac tonic
*Epilobium augustifolium *L. (Onagraceae) not collected	fireweed	aerial parts	--
*Equisetum palustre *L. (Equisetaceae) JB60	horsetail	aerial parts	minerals
*Galium aparine *L. (Rubiaceae) JS107	cleavers	aerial parts	coats
*Gaultheria shallon *Pursh. (Ericaceae) JS100	salal	aerial parts	--
*Holodiscus discolor *(Pursh.) Maxim. (Rosaceae) JB5	Ocean spray	aerial parts	gives sweet flavour to the milk
*Hypochaeris radicata *L. (Asteraceae) JB11	Hairy cats ear	Stems	--
*Lactuca muralis *(L.) Fresen. (Asteraceae) JB23	Wall lettuce	aerial parts	--
*Linum usitatissimum *L. (Linaceae) not collected	flax	Seeds	coat
*Lonicera caprifolium *L. (Caprifoliaceae) not collected	honeysuckle	aerial parts	--
*Malus *spp. (Rosaceae) not collected	Apple	Pulp	--
*Melissa officinalis *L. (Lamiaceae) JS006	Lemon balm	aerial parts	calming
*Origanum *sp. (Lamiaceae) JS003	oregano	aerial parts	--
*Phalaris arundinacea *L. (Poaceae) JB30	Reed canary grass	aerial parts	--
*Plantago major *L. (Plantaginaceae) JB62a	plantain	aerial parts	--
*Pseudotsuga menziesii *(Mirbel) Franco (Pinaceae) JS049	Douglas fir	young or thin branches	--
*Rosa nutkana *K. Presl (Rosaceae) JS013	Nootka rose	Aerial parts	--
*Rosa gymnocarpa *Nutt. (Rosaceae) JS044	Baldhip, native and domestic rose and rose hips	aerial parts	--
*Rubus idaeus *L. ssp. *idaeus *(Rosaceae) not collected	raspberry	aerial parts	--
*Rubus idaeus *L. ssp. *idaeus *(Rosaceae) not collected	raspberry	aerial parts	--
*Rubus parviflorus *Nutt. (Rosaceae) JB25	thimbleberry	aerial parts	--
*Rubus *discolor Weihe & Nees JS028, *Rubus ursinus *Cham. & Shlecht. JS115 and *Rubus laciniatus *Willd. (Rosaceae) JB55	blackberry	Branches	--
*Rubus spectabilis *Pursh (Rosaceae) JB 038	Salmonberry	aerial parts	--
*Rumex acetosella *L. JS047	Sheep sorrel	aerial parts	--
*Salvia officinalis *L. (Lamiaceae) JS035	Sage	aerial parts	--
*Sambucus racemosa *L. (Caprifoliaceae) not collected	red elderberry	aerial parts	--
*Sonchus arvensis *L., (Asteraceae) not collected	Sow thistle	aerial parts	--
*Stellaria media *(L.) Cyrill. (Caryophyllaceae) JS108	chickweed	aerial parts	--
*Taraxacum officinale *(L.) Weber (Asteraceae) JB96	dandelions	aerial parts	--
*Thuja plicata *Donn ex D. Don (Cupressaceae) JBR 21	red cedar	inner bark and fronds	--
*Thymus vulgaris *L. (Lamiaceae) JB61, JB73	thyme	aerial parts	--
*Tsuga heterophylla *(Raf.) Sarg. (Pinaceae) JB113	hemlock	aerial parts	maintain body heat
*Urtica dioica *L. (Urticaceae) JS023	nettles	Aerial parts	tonic
*Vaccinium membranaceum *Dougl., *Vaccinium parvifolium *Smith (Ericaceae) JS045	huckleberry	Foliage, berries	carotene, manganese, energy

**Table 3:** Plants considered poisonous to ruminants in British Columbia

*Digitalis *sp. (Scrophulariaceae)	Foxglove	plant
*Narcissu*s sp. (Amaryllidaceae)	Daffodils	plant
*Rheum officinale *(Polygonaceae)	Rhubarb	leaf
*Rhododendron *sp. (Ericaceae)	Rhododendron	plant
*Euphorbia *sp. (Euphorbiaceae)	Spurge	plant
*Cytisus laburnum *(Leguminosae)	Laburnum	seeds
*Wisteria *sp. (Leguminosae)	Wisteria	plant
*Taxus *sp. (Taxaceae)	Yew	tree
*Delphinium *sp. (Ranunculaceae)	Larkspur	plant
*Solanum dulcamara *(Solanaceae)	Nightshade	plant
*Lupinus *sp. (Papilionaceae)	Lupine	plant

**Table 4:** Plants used as pregnancy feeds for ruminants in British Columbia

*Fucus vesiculosus *L. (Fucaceae; Brown Algae) JBCL 11	Kelp	plant
*Rubus idaeus *L. ssp. *idaeus *(Rosaceae) not collected	raspberry	leaves
*Urtica dioica *L. (Urticaceae) JS023	Nettles	plant
*Taraxacum officinale *(L.) Weber (Asteraceae) JB96	dandelions	leaves & flowers
*Pseudotsuga menziesii *(Mirbel) Franco (Pinaceae) JS049	Douglas fir	branches
*Tsuga heterophylla *[Raf.] Sarg. (Pinaceae) JS113	Western hemlock	branches
*Salix lucida *Muhl. (Salicaceae) not collected	Willow	branches
*Epilobium augustifolium *L. (Onagraceae) not collected	Fireweed	plant
*Pisum *sp. (Fabaceae) not collected	Pea vines	plant
*Taraxacum officinale *(L.) Weber (Asteraceae) JB96	dandelions	plant
*Lonicera involucrata *(Richards.) Banks ex Spreng (Caprifoliaceae) not collected	black Siamese-twinberry	plant
*Rubus idaeus *L. (Rosaceae) not collected	Wild raspberry	leaves

### Various injuries – abscess

A root decoction of Oregon grape (*Berberis aquifolium*/*Mahonia aquifolium*) or root decoction of Echinacea (*Echinacea *spp.) is given as the drinking water for seven to ten days. Ruminants are also feed ample amounts of fresh or dried comfrey (*Symphytum officinale*).

#### Cuts, scratches

Calendula (*Calendula officinalis*) infused oil is considered beneficial for the reversal of numerous skin and tissue conditions. It is used only after the threat of infection has passed. It is not used on deep wounds since it is felt that calendula may seal the wound too quickly preventing drainage. It was claimed that olive oil does not work on cows as an ointment since it does not absorb into the skin; lanolin does.

Chewed up leaves of yarrow (*Achillea millefolium*), are put on wounds and then wrapped with breathable tape. The spore mass of puffball (*Bovista pila*, *Bovista plumbea*) is applied to hoof trimming 'nicks' that bleed excessively. It is then wrapped with breathable first-aid tape. Comfrey (*Symphytum officinale*) and calendula (*Calendula officinalis*) are used on injuries only after the threat of infection has passed (see wounds).

#### Dehorning adult animals

After horns are sawed off, the wound area is cauterized with a hot iron to deaden the pain. Once the initial bandages have been removed (after two days), the cavities are packed with *Usnea *lichen to enhance the healing process.

#### Dehorning – disbudding

Disbudding of young kids is done with a hot iron. If the scab left after disbudding is knocked off and excessively bleeds, dried puffball (*Bovista pila*, *B*. *plumbea*) sporemass is applied to the wound which is then bandaged if possible. Clean puffball spores (*Bovista pila*, *B. plumbea*) are dusted on wounds left from removing loose scurs (horns) or small horn regrowths.

#### Proud flesh

Goats are treated for proud flesh with several herbs. Wound-knitting herbs (comfrey – *Symphytum officinalis*, goldenseal-*Hydrastis canadenis *or calendula – *Calendula officinalis*) are not used on fresh wounds since they are thought to close the wound too quickly, before it has healed underneath. Bee propolis is also used as a wound treatment Proud flesh is dealt with by scrubbing until it bleeds twice a day with a stiff scrub brush. Then hydrogen peroxide is applied using a syringe. A purchased product called 'Wonder Dust' antifungal powder is sprinkled on the wound. Once the wound is healed vitamin E, and infused oil or salve of St. John's Wort (*Hypericum perforatum*) or essential oil of lavender (*Lavandula officinalis*) is put on the area. Another treatment involves a comfrey poultice (*Symphytum officinalis*) made with 1 tsp curcumin or fresh grated turmeric and bromelain (crush 1 or 2 purchased pineapple or papaya enzyme tablets for papain).

#### Sternal abscess

The gleba (sporemass) of *Bovista pila *or *Bovista plumbea *is applied to wounds. Alternate applications are made with the salve recorded below or with poultice of yarrow, or a combination of them both is used to draw out the pus. A salve is made with 1/2 cup honey or sugar, 1/2 cup alum, 1 vitamin C pill (or ascorbic acid powder) and 1/2 cup ground *Usnea *spp. (old man's beard lichen).

#### Deep wounds, broken horn, shearing cut, wire cut

Wounds are bathed with a slimy tea made of mallow (*Malva *sp.) (3 tsp mallow aerial parts steeped for 15 minutes with 1 cup of boiling water). Another treatment consists of the infused oil of St. John's Wort (*Hypericum perforatum*) (2 cups of olive oil and 1 1/2 oz (50 g) *Hypericum *flowers in a glass jar, stored in the dark for 2 months before straining and using). Another treatment consists of a wad of clean spider web put on the bleeding wound. Cornstarch is sprinkled on the wound to help blood clot.

Another treatment consists of a wash made with an infusion of 2 tsp dried aerial parts of self heal (*Prunella vulgaris*) steeped in 1 cup of boiling water and allowed to cool. Ample fresh or dried comfrey aerial parts are fed. To boost the immune system and fight infection, Echinacea or Oregon grape teas are given for seven days. These are made with 1/2 cup coarsely cut dried Echinacea or Oregon grape roots simmered in water for 10–15 minutes. One cup of tea is diluted in 1 gallon of water and given as the only drinking water.

*Bovista pila *or *Bovista plumbea *puffball gleba (sporemass) is applied to a clean wound to stop bleeding. A chewed leaf of yarrow (*Achillea millefolium*) is used as a poultice to staunch bleeding on a superficial wound. Leaves of shepherd's purse (*Capsella bursa*-*pastoris*) can be used instead of yarrow.

#### White line abscess or foot rot

After paring out the rot, a zinc-based or copper-based liquid is put into the pared-out pocket, with old man's beard lichen (*Usnea *spp.) inserted into cavity to hold the liquid in. If the animal is lame (pus pockets forming) it is treated with penicillin for three to four days. Copper-based liquids are not used for sheep.

#### Wounds – bruises

Wild arnica (*Arnica *sp.) leaves or flowers (1 or 2) are rubbed on to bruises or the crushed leaves are bandaged on the wound. Arnica is not used on open wounds. Arnica is only used externally (or as a homeopathic drug). Ointments containing bee propolis and other bee products are used to seal wounds and protect them against flies. Pine tar is used to seal wounds and keep flies out.

#### Management – Bedding

Big leaf maple leaves (*Acer macrophyllum*) are used as bedding to ensure that grass seeds do not get into the compost. These leaves are raked up and stored dry in autumn.

#### Flies

The same fly control remedies are used on all ruminants. Bunches of vanilla leaf (*Achlys triphylla*), European rue leaves (*Ruta graveolens*) or European pennyroyal (*Mentha pulegium*) are hung in stables and the milking room. These are kept out of the animals' reach as some are mildly poisonous. Animals are rubbed with oil that has European pennyroyal (*Mentha pulegium*) soaked in it. This is not used on pregnant animals. Lavender (*Lavandula officinalis*), cloves (*Eugenia caryophyllata*) and peppermint (*Mentha piperita*) essential oils are dissolved in water and used for fly control. Citronella is also used for fly control.

#### Flystrike (maggot infestation)

All ruminants are treated for flystrike with comfrey salve, if the wound is partially healed or if it is not deep. Pine tar is applied if it is warm weather (corresponding to the fly season).

### Caprine arthritis

Turmeric powder (*Curcuma longa*) (1 tsp to 1 tbsp depending on the animal's weight) is added daily to moist food. Results are seen in two – three weeks. Goats are given cut branches of native willows such as Scoulers willow (*Salix scouleriana*) or Pacific willow (*Salix lucida *spp *lasiandra*).

#### Pre-show protection

An Echinacea (*Echinacea *spp.) tincture is given to animals before shows. It consists of 4 ounces of dried *Echinacea purpurea *or *augustifolia *root or 1 or 2 fresh *Echinacea *chopped roots. A jar or glass bottle is half-filled with the chopped fresh or dried root. Vodka, brandy or rum is added until it covers the root completely. This is stored in a dark place for two to eight weeks. It is shaken daily for the first week then weekly for the remaining weeks. Then it is decanted into a tincture bottle. One tsp of Echinacea (*Echinacea purpurea *or *augustifolia*) tincture per animal in is added to the feed bowl daily for self-medication (immune stimulant) at least six to ten days before the show. A by-product from processed Echinacea can be used instead of a purchased product to reduce costs. Nettles (*Urtica dioica*) are fed daily for a few weeks before the show.

#### Pain killer

Catnip (*Nepeta cataria*) or valerian (*Valeriana officinalis*) are used as pain killers for goats. One tbsp of chopped valerian root is steeped in 1 cup of hot water for 20 minutes. The pot is covered to retain the essential oils. Or 1 tbsp of chopped catnip herb is put in 1 cup of hot water and steeped for 10 minutes. Or willow twigs (*Salix *sp.) are given since they contain salicin.

#### Urine scald

Propolis cream (propolis, beeswax, shea butter), or any barrier salve are used on sheep with urine scald.

### Various health issues – CAE (Caprine arthritis encephalitis)

The following treatments are given as palliatives only. Powdered turmeric (*Curcuma longa*), 1/2 tbsp per day, is mixed into the food. This is said to prolong the life of the animal and add to its comfort. Finely chopped branches of native willow (*Salix *sp), Scoulers willow (*Salix scouleriana*) and Pacific willow (*Salix lucida *spp. *lasiandra*) are added to the food.

#### Deformed kids (case history)

A doe had produced kids with front limb deformities two years in a row (from different sires). The owner speculated that the doe had been eating mouldy bits of hay that other goats refused during early pregnancy. Therefore during the subsequent pregnancy, the owner regularly fed the doe turmeric with the result that the doe gave birth to completely normal triplets. The dose was 1/2 tbsp turmeric (*Curcuma longa*) added daily to the feed three weeks prior to breeding and for at least a full month after breeding to 'detoxify' the system of the doe. The owner repeated the treatment the following year during pregnancy with the same result – normal triplets.

#### Respiratory conditions

Goats are allowed to browse on mullein (*Verbascum thapsus*) as a respiratory tonic (self-medication). Several crushed cloves of garlic are given orally as an antibiotic for goats that aren't milking. A strong tea (decoction) of Oregon grape root (*Berberis aquifolium*/*Mahonia aquifolium*) or Echinacea root (*Echinacea purpurea *or *Echinacea augustifolia*) is given as the only source of drinking water (1/2 cup of coarsely cut dried Oregon grape root or Echinacea root in 2.5 cups of water, simmered for 10 to 15 minutes). One cup of the resulting fluid is diluted with 1 gallon of water and given as the drinking water.

#### Unidentified sickness

The animal had the following symptoms: low energy, tail down, stressful bleat, separated itself from herd, was hunched, had difficulty lying down (and other symptoms). It was given whole leafy branches of blackberry (*Rubus ursinus *and *laciniatus*), grape (*Vitis *sp.), and willow (*Salix *sp), free choice.

#### Urinary stones

Sheep and goats with urinary stones are given 1/3 cup apple cider vinegar twice a day diluted in 1 cup of water, orally.

### Diarrhoea, scours

A combination of fresh plantain leaves (*Plantago *sp.), flower heads of calendula (*Calendula officinale*), tops of nettles (*Urtica dioica*) and leaves of comfrey (*Symphytum officinale*) was given. If blood was seen in the stool, 1/2 tbsp of slippery elm bark powder (*Ulmus fulva*) was added. Calendula (*Calendula officinalis*) flower head tea is given to calves with sore stomachs.

Branches of long needle yellow pine (*Pinus ponderosa*) are put in the pen of young animals (four weeks old, still nursing) with grey pasty diarrhoea. They can then eat it free choice. Animals will self-medicate with aerial parts of fresh cinquefoil (*Potentilla *sp). An alternative treatment consists of a drench made with 1 part or 1 tsp marshmallow (*Althaea officinalis*), 1/2 part dill seed (*Anethum graveolens*), 1 part bark of white willow (*Salix *sp) and 1 part inner stem bark of slippery elm (*Ulmus fulva*). If not already powdered it is ground and mixed with water before drenching. A pinch of cinnamon (*Cinnamomum zeylandica*) and a pinch of ginger (*Zingiber officinalis*) can be added. If there is blood in the feces then 1/4 part cloves (*Syzygium aromaticum*) is added to control coccidia. A dose of 2 tbsp is used for animals over 50 lbs. A dose of 1 tbsp is used for animals under 50 lbs. The drench is given once a day until the diarrhoea stops (two to three days). Goats are allowed to self-medicate with the charcoal from a cold wood fire. Animals are starved for one day, then purged with a senna pod infusion (*Senna *sp.). Afterwards they are drenched with slippery elm (*Ulmus fulva*) powder to soothe the stomach.

### Eye problems (Conjunctivitis)

Infected eyes of cows are treated with eyebright tea (*Euphrasia officinalis*) which is applied several times by soaking gauze and dropping the tea onto the eyes. Alternatively a tea made with a chamomile (*Matricaria chamomilla*) tea bag is allowed to cool, then the teabag is dipped back in the tea and a few drops of tea are dropped into the eye of the animal.

### Parasites – Internal parasites (endoparasites)

The following are blended together: 5 leaves of wormwood (*Artemisia *sp.), a handful of sunflower seeds (*Helianthus annuus*), a couple of fresh minced or crushed garlic cloves (*Allium sativum*), left-over onion and skins and honey to sweeten. The mixture is fed once a week as a preventative. Dried ground nettles seeds added to feed (*Urtica *sp.) are given free choice. Limited results are seen from 3–4 fresh minced cloves of garlic and tops added to the feed.

Conifers fed free choice are said to prevent worms. Douglas fir (*Pseudotsuga menziesii*), red cedar (*Thuja plicata*) and juniper (*Juniperus communis*) are given. Common juniper berries (*Juniperus communis*) are said to be effective against liver fluke. Alternatively each goat gets 1/2 tsp wormwood (*Artemisia *sp.) in its feed. This treatment comes from an owner who uses wormwood (*Artemisia *sp.) infrequently and whose goats do not like it. Branches of the following are fed in winter time: cedar (*Thuja plicata*), Douglas fir (*Pseudotsuga menziesii*), snowberry (*Symphoricarpos albus*). Alternatively goats are given 2 drops of tea tree oil (*Melaleuca alternifolia*) on the tongue at milking time. This does not cause an off-taste in the milk. Goats are allowed to self medicate on long needle yellow pine (*Pinus ponderosa*). Animals are fed armfuls of carrot (*Daucus carota*), celery (*Apium graveolens*), parsley (*Petroselinum *sp.) or parsnip tops (*Pastinaca sativa*).

#### Coccidiosis

Feeding ample amounts of branches of Douglas fir (*Pseudotsuga menziesii*) is said to prevent coccidia.

#### External parasites – Lice

Bark shavings of cedar (*Thuja plicata*) are put in the bedding. Powdered neem (*Azadirachta indica*) is brushed into the coat. Neem is used less often than clipping. Alternatively the infused oil of pennyroyal (*Mentha pulegium*) is rubbed onto the top of the head and the spine of the goat – it is brushed well into the coat.

### Dairy issues – Mastitis

Goats and sheep with mastitis are given one-third cup of apple cider vinegar diluted in water twice a day. A tea of yarrow (*Achillea millefolium*), honey, sea salt, burdock root (*Arctium *sp.) and white willow bark (Salix sp.) is given. It is made with 1/3 cup of yarrow (whole chopped plant with flowers), 1/3 cup chopped burdock root and 1/3 cup chopped white willow bark. Three cups of boiling water are poured over the herbs and steeped for 15 – 20 minutes. Sea salt and honey is added. When cool, the herbs are applied as a poultice, or a cotton cloth is dipped in the warm infusion and put around the udder until the poultice cools.

#### Mastitis

Cows with mastitis have apple cider vinegar (1/2 cup) added to the grain and fed twice a day. Cows are treated only if they show susceptibility. Woodsage (*Teucrium scorodonia*) tincture is infused in the udder. An infusion of cleavers (*Galium aparine*) is made by steeping 1 tbsp of cleavers in 1 cup of boiling water for 15 minutes. This is then drenched to help boost circulation in the udder and for lymph support.

#### Milk production

Pregnant and lactating goats and cows are allowed access to fresh nettles or wilted cut nettles. Milking ewes are given a tea of dill seed for milk production. Dill seed (*Anethum graveolens*) (2 tsp) is steeped in 1 cup of boiling water for 10–15 min. Or 1/2 cup dill seeds is steeped in water overnight. This is then boiled until very dark in color and strained. Each animal is given 1 cup of this dill tea per day as the drinking water. Armfuls of comfrey (*Symphytum officinale*) are reputed to increase butterfat and act as a laxative. A handful of fresh or dried leaves of thornless raspberry (*Rubus *sp.) is given free choice.

#### Udder edema

A handful of dandelions (*Taraxacum officinale*) leaves and/or cornsilk (*Zea mays*) are fed as diuretics. Both can be dried (on a cookie sheet on low heat -100 to 200 degrees- in the oven) and used in the winter. Fresh or dried comfrey (*Symphytum officinalis*) leaves and/or stems are also fed.

#### Milk reduction (drying off)

Goats are dried off using a paste of 1 tsp of dried sage (*Salvia *sp.) in water. The paste is put on the udder. Alternatively the tsp of dried sage is fed by crumpling it on grain with molasses for palatability. A couple of stalks of comfrey (*Symphytum officinale*) are given every couple of days during the lactation period.

### Diet

Sheep are fed kelp (1 tsp per animal for two weeks), three times a year to keep their coats healthy. One tbsp of bee pollen fed by hand daily is said to keep sheep tame and healthy. Sheep eat aerial parts of the following species: nootka rose (*Rosa nutkana*), blackberry (*Rubus *sp.), raspberry (*Rubus idaeus*), yarrow (*Achillea millefolium*), oregano (*Origanum *sp.), thyme (*Thymus *sp.), sage (*Salvia *sp.) and tarragon (*Artemisia dracunculus*).

Goats are allowed to browse resinous plants in winter to help them maintain body heat: red alder (*Alnus rubra*), fresh and dried leaves of arbutus (*Arbutus menziesii*), grand fir (*Abies grandis*), hemlock (*Tsuga *sp.), young or thin branches of Douglas fir (*Pseudotsuga menziesii*), inner bark and fronds of red cedar (*Thuja plicata*), inner bark of big-leaf maple (*Acer macrophyllum*). Western yew (*Taxus canadensis *or *Taxus brevifolia*) is eaten without problems by goats, deer and moose. If goats are stall-fed, they are given a variety of branches, clean weeds, and fruit/vegetable trimmings. They are fed apple pulp (*Malus *sp.), chopped-up pumpkin (*Cucurbita pepo*-vitamin A), and clean fruit/vegetable scraps from the kitchen.

Goats relish the following: thistle (*Cirsium arvense*), blackberry branches (*Rubus ursinus *and *Rubus laciniatus*), burdock (*Arctium minus *or *Arctium lappa*), canary grass (*Phalaris canariensis*), cleavers (*Galium aparine*) (helps coats), chicory (*Cichorium intybus*), crepis (*Crepis capillaris*), dandelions (*Taraxacum officinale*), fireweed (*Epilobium augustifolium*), hairy cats ear (*Hypochaeris *sp.) (stems especially), honeysuckle (*Lonicera caprifolium*) and huckleberry (*Vaccinium membranaceum*, *Vaccinium parvifolium*). Nettles (*Urtica dioica*) are used as a tonic. To accustom animals to nettles it is given dried and ground in feed first, then wilted, finally it is given fresh.

Goats will also browse miners lettuce (*Claytonia perfoliata*), ocean spray (*Holodiscus discolor*) (said to give a sweet flavour to the milk), pearly everlasting (*Anaphalis margaritacea*), plantain (*Plantago *sp.), raspberry (*Rubus idaeus*), red elderberry (*Sambucus racemosa*), red osier dogwood (*Cornus sericea*) especially in winter; native and domestic rose and rose hips (*Rosa *sp., *Rosa nutkana*), salal (*Gaultheria shallon*), Oregon grape (*Berberis aquifolium*/*Mahonia aquifolium*), salmonberry (*Rubus spectabilis*), sheep sorrel (*Rumex acetosella*), silver-green/pathfinder (*Adenocaulon bicolor*), thimbleberry (*Rubus parviflorus*), vanilla leaf (*Achlys triphylla*), and weeds such as lamb's quarters (*Chenopodium album*), chickweed (*Stellaria media*), sow thistle (*Sonchus arvensis*), wall lettuce (*Lactuca muralis*) and yarrow (*Achillea millefolium*). Lemon balm (*Melissa officinalis*) is given as a calmative.

#### Trace & other minerals

Sunflower seeds are fed with the shells to add the calcium needed for growing kids, and pregnant and lactating does. Washed (sand-free) seaweeds fresh from the sea, such as bladderwrack are given to provide iodine and trace minerals. Flax (*Linum usitatissimum*) whole seed (milder taste) is fed to improve the coat. One tbsp is given with each feeding of grain. Goats search for horsetail (*Equisetum arvense*) in spring. Twelve goats (one pen) are given 6 dried horsetail plants (*Equisetum arvense*) or they are given it fresh once or twice a month (free choice). Dried nettles (*Urtica dioica*) are sprinkled on the food daily or when available. A handful of dry dandelions leaves (*Taraxacum officinale*) is given every week when available. Kelp, a 3-litre pail for 90 cows, is put into the bottom of the hay manger so that the cows have "free choice" access to that much each day.

### Pregnancy

Ruminants are fed kelp to provide trace minerals and improve fertility. At each feeding 200 goats are given 1/2 cup kelp or they are given 1 cup/day. Kelp is fed more often in winter to reduce costs. The quantities are not increased otherwise the milk will test positive for iodine. One or 2 tbsp brewers' yeast mixed in with the kelp helps rumen bacteria. If sheep are fed (grain) consistently by 9 a.m. during the pregnancy, it is said that they will lamb in the daytime.

Fresh raspberry leaves (*Rubus idaeus*) are uterine tonics and can be given before the dams are bred. Dried, stored leaves are also used. If ample amounts are available they are fed as hay in late pregnancy to tone the uterine muscles. Alternatively 1 tbsp of the leaves is put on top of the grain daily two to three weeks before kidding or lambing. A postpartum supplement consists of 1/2 tbsp of raspberry leaves daily. Blackberry and raspberry leaves (*Rubus *spp.), branches of Douglas fir (*Pseudotsuga menziesii*) and Western hemlock (*Tsuga heterophylla*) are fed during pregnancy for their vitamin C content. Molasses is fed to prevent pregnancy-related ketosis and the selenium in the diet is increased. Pregnant and lactating goats and cows are given access to fresh or wilted nettles (*Urtica dioica*) and fresh leaves and flowers of dandelions.

Red cedar (*Thuja plicata*) is fed if the animals are deficient in copper (this treatment is not specific to pregnancy. Large amounts of red cedar (*Thuja plicata*) are not given in early pregnancy (first six weeks) because of a neurotoxin in the plant. Red cedar (*Thuja plicata*) makes the milk pitchy flavoured. Shore pine (lodgepole pine or jackpine) (*Pinus contorta*) may cause abortion. Goats like to nibble broom (*Cytisus scoparius*) which can act as a cardiac tonic but they are not given broom during pregnancy.

Fresh air, sunshine and exercise are used to help the animals give birth. Hay or bundles of weeds are thrown on the snow so that they have to plough through the snow for them. During the summer, green plants such as willow (*Salix *sp), fireweed (*Epilobium *sp.), pea vines (*Pisum *sp.), dandelions (*Taraxacum officinale*), black Siamese-twinberry (*Lonicera involucrata*) and wild raspberry (*Rubus *sp.) are cut and dried in bundles. During the winter, when animals are pregnant, a bundle is fed every Sunday.

#### Pregnancy toxaemia – ketosis

Animals are hand fed all and any tasty forest browse (e.g. salal (*Gaultheria shallon*), huckleberry (*Vaccinium *sp) or armfuls of comfrey (*Symphytum officinale*).

#### Retained placenta

A handful of leaves of English ivy (*Hedera helix*) is fed at the time of birth, to contract the uterus, and prevent retained placenta. A tincture of lady's mantle (*Alchemilla vulgaris*) (90 ml twice a day (after evaporating off the alcohol) is given for uterus infection after calving, diarrhoea or for retained placenta. Alternatively it was given as a drench for five days. There are reports that cows eating *Alchemilla vulgaris *have tainted milk.

## Discussion

The non-experimental validation of the plants is provided in Table 5. The plants are listed in alphabetical order. As stated previously this validation process was undertaken in the process of preparing the draft manual of remedies and continued after the workshop when the final version of the manual was prepared.

**Table 5:** Non-experimental validation of plants used for ruminants in British Columbia

Medicinal plant	Validation information	Reference
*Acer macrophyllum*	*Acer macrophyllum *young shoots were eaten raw in spring by the Thompson Indians. The bark slips off easily at that time. The leaves of *Acer saccharum *contain less than 2% percent calcium, 0.24 percent magnesium, 0.75 percent potassium, 0.11 percent phosphorus, 0.67 percent nitrogen, and 11.85 percent ash (dry weight). Mid level validity as bedding.	10, 13, 14
*Achillea millefolium*	Achilles reportedly staunched the wounds of his soldiers with this plant thus providing the name of the genus *Achillea*. *Achillea millefolium *is also used traditionally as an *emmenagogue*. An *in vitro *assay using the crude extract of the aerial parts of *A. millefolium *showed estrogenic activity. Apigenin and luteolin are reportedly the most important estrogenic compounds. The aqueous extract of *Achillea millefolium *(0.3–1.2 g/kg, p.o./day) was effective in protecting the gastric mucosa of male and female Wistar rats against acute gastric lesions induced by ethanol and indomethacin and in healing chronic gastric lesions induced by acetic acid with (ED(50) = 32 mg/kg, p.o.). Mid level validity for all uses.	9, 16, 17
*Achlys triphylla*	The use of this plant as a fly repellent is Native American in origin. Four new flavonol glycosides were isolated from the underground parts of *Achlys triphylla *in addition to eight known compounds. Mid level validity as a fly repellent	8, 15
*Alchemilla vulgaris*	Lady's mantle has the nickname, "a woman's best friend", and this is reflected in the ethnoveterinary use for retained placenta. Extracts from *Alchemilla vulgaris *L. inhibited 50% of the activity of porcine pancreas elastase at concentrations of 0.16 mg/ml, against a synthetic substrate. This study claimed a possible role by the extract in the protection of conjunctive and elastic tissues adversely affected by proteolytic enzymes. Mid level validity for retained placenta.	12, 18
*Allium cepa*	*Allium cepa *oil given at 5 mg/kg body weight/day for 2 weeks showed anthelmintic activity in rats experimentally infected with *Trichinella spiralis *with a decline in the adult worms and muscle larvae. It was less effective as a prophylactic treatment prior to *Trichinella spiralis *infection. Mid level validity for endoparasites.	19
*Allium sativum*	Experiments with the intestinal parasite *Entamoeba histolytica *have shown that pure allicin inhibits both the cytopathological effects associated with infection and the growth of the parasite by blocking its cysteine proteases. Other studies with allicin have shown that it has inhibitory effects on a wide range of bacteria, on some fungi and on a few protozoans. Mid level validity for all uses.	20
*Allium sativum*	Treatment with garlic extract has been shown to activate macrophages, and suppress lesion growth in *L. major *infected mice. A garlic extract, showed no significant effect in the reduction of *L. chagasi *parasite load. The maximal survival of the garlic treated animals, despite their high parasitic burden, might be explained by a mild non-specific protective effect of the garlic treatment. In a *L. major *model, garlic treatment was more effective than chemotherapy with the first line drug glucantime, showing an additive effect with the antibiotic. There may be a protective effect of garlic treatment if administered previous to infection, in an immunoprophylactic vaccination schedule against visceral leishmaniasis. Mid level validity for all uses.	21
*Althaea officinalis*	Originally from China, this plant was an ingredient in the original marshmallows eaten by Egyptians and Romans. Over 1000 species in the Malvaceae family contain healing mucilage. The methanol extract of *Althaea officinalis *roots was active against *P. gingivalis*, *Prevotella *spp. and *Actinomyces *spp. (9 of 12 strains had a MIC ≤ 3125 mg/L. The decoction had higher MIC values (4096–8192 mg/L. The strains of *C. gingivalis*, *V. parvula*, *E. corrodens *and *Peptostreptococcus *spp. were inhibited by an MIC = 8192 mg/L, those of *F. nucleatum *by an MIC ≥ 16384 mg/L. Mid level validity for diarrhea.	12, 22
*Anethum graveolens*	The ancient Egyptians and Greeks recorded the medicinal value of dill. The monoterpene carvone is a major constituent (50%–60%) of the essential oil. This monoterpene has a calming effect and is used in gripe water preparations. Falcarindiol exhibited the greatest activity of the three active principles isolated from the whole herb of *Anethum graveolens *with minimum inhibitory concentration (MIC) values in the range 2–4 μg/mL against mycobacteria (*Mycobacteriumfortuitum*, *Mycobacterium phlei*, *Mycobacteriumaurum *and *Mycobacterium smegmatis*). Plant compounds oxypeucedanin and oxypeucedanin hydrate also showed moderate anti-mycobacterial activity against the same mycobacteria with MIC values in the range 32–128 μg/mL. Mid level validity for diarrhea.	12, 23, 24
*Apium graveolens*	The Greeks recorded the medicinal value of wild celery. The ascaricidal efficacy of the oil of *Apium graveolens *tested *in vitro *against the eggs and larvae of *Ascaris lumbricoides *was less effective than the aqueous extracts of 1% *Artemesia *and 5% of *Albizzia *and *Inula*. Mid level validity for endoparasites.	12, 25
*Arctium lappa*	*Arctium lappa *has anti-bacterial and antifungal activity, diuretic, anti-oxidant and anxiolytic action, a platelet anti-aggregating effect and HIV-inhibitory action. *Arctium lappa *constituents inhibited the tested endodontic pathogens *Enterococcus faecalis*, *Staphylococcus aureus*, *Pseudomonas aeruginosa*, *Bacillus subtilis *and *Candida albicans*. A previous study on three forms of the rough extract of this plant (20% tincture, extract concentrated by rotaevaporation and lyophilized extract), found that the lyophilized extract was the most effective against *B. subtilis *and *C. albicans*. Mid level validity for mastitis.	26
*Arnica *sp.	*Arnica montana *is indigenous to Central Europe. The methanol extract of *Arnica montana *flowers had a better antibacterial activity than the decoction (with MICs two or three times lower). The inhibiting concentrations of the methanol extract against *P. gingivalis *(3 of 5 strains), *Prevotella *spp., *E. corrodens*, *Peptostreptococcus *spp. and *Actinomyces *spp. had acceptable values (MIC ≤ 2048 mg/L) for the use in mouthwashes for the correct hygiene of the oral cavity. *C. gingivalis *and *V. parvula *(MIC 4096 mg/L) were less sensitive and so was *F. nucleatum *(MIC 16384 mg/L). Mid level validity for wounds.	9, 22
*Artemisia *sp., *Artemisia*	Tarragon leaves are rich in iodine, minerals and vitamins C and A. This study compared the *in vitro *and *in vivo *anthelmintic activity of *Artemisia brevifolia *with levamisole. *In vitro *studies revealed anthelmintic effects of crude aqueous (CAE) and methanol extracts (CME) of *Artemisia brevifolia *(whole plant) on live *Haemonchus contortus *as evident from their paralysis and/or mortality at 6 h post exposure. For *in vivo *studies, the whole plant of *Artemisia brevifolia *was administered as crude powder (CP), CAE and CME at graded doses (1, 2 and 3 g kg(-1) body weight (b.w.) to sheep naturally infected with mixed species of gastrointestinal nematodes. Maximum reduction (67.2%) in eggs per gram (EPG) of faeces was recorded on day 14 post treatment in sheep treated with *Artemisia brevifolia *CAE at 3 g kg(-1) b.w. Levamisole produced a 99.2% reduction in EPG. However, increase in EPG reduction was noted with an increase in the dose of *Artemisia brevifolia *administered as CP, CAE and CME. Mid level validity for endoparasites.	12, 27
*Azadirachta indica*	Groups of 11–12 angora goats were treated with an azadirachtin-rich extract of neem seeds with an azadirachtin concentration of 650 ppm or 125 ppm, with Neguvon((R)), or untreated (control). Their louse burden (*Damalinia limbata *Phthiraptera) was assessed for 22 weeks. A reduction in louse densities of 76–96% was observed from week 2 to week 18 after treatment with the neem solution containing azadirachtin at a concentration of 650 ppm. At the lower test concentration (125 ppm) a reduction of 60–92% was recorded from week 2 to week 14. The extract reduced the survival of both adult and nymph stages of *Damalinia limbata*. Mid level validity for ectoparasites.	28
*Berberis aquifolium*/*Mahonia aquifolium*	Berberine is an isoquinoline alkaloid that has been isolated from *Berberis aquifolium *(Oregon grape), *Berberis aristata *(tree turmeric) and *Berberis vulgaris *(barberry). It has antibiotic, antitumor and antidiarrheal activities. Berberine may have multiple effects on the cardiovascular system. Mid level validity as a respiratory tonic and for wounds.	29-32
*Blechnum spicant*	Low level validity for magnesium imbalance but the plant is reported to grow in magnesium rich soil.	33
*Bovista pila*	The basidiomycete *Bovista *sp contains psathyrellon B. The hexacyclic metabolite bovistol exhibited very weak antibacterial (MIC *Micrococcus luteus *100 μM) and antifungal (MIC Mucor miehei 100 μM) activities. Mid level validity for wounds.	34
*Bovista plumbea*	Puffball has been traditionally used to stem bleeding and promote healing. Penicillin acylase (penicillin amidohydrolase, EC 3.5.1.11) was isolated in the basidiomycete *Bovista plumbea*. Mid level validity for wounds.	35
*Calendula officinalis*	Culpepper describes *Calendula *flowers as a "comforter of the heart and spirits". The methanol extract of *Calendula officinalis *flowers had antibacterial activity; it inhibited *Actinomyces *spp. at MICs ≥ 8192 mg/L. Mid level validity for wounds and diarrhea.	9, 22
*Capsella bursa*-*pastoris*	Culpepper recorded the use of the European plant *Capsella *for wounds. *Capsella bursa-pastoris *is included in the VIIIth and IXth editions of USSR pharmacopoeia and is an official remedy in other countries. It is used for uterine bleeding, malignant ulcers and cancer of the stomach, tumors, uterine cancer and fibroma, and for all types of kidney bleeding and diseases in homeopathy. Extracts of leaves and roots contain neutral lipids (62.6 and 58.5%), glyco-(20.8 and 17.8%), and phospholipids (16.6 and 23.7%, respectively). The seed oil contains fatty acids (FA) up to 50% linolenic and ~1% erucic acid. Beta-carotene and beta-sitosterol were identified in the aerial part. Mid level validity for wounds but more data is needed.	9, 36
*Cinnamomum zeylanicum*	An anecdotal report described the resolution of *Salmonella *in a chronic carrier by the use of cinnamon. There are other reports that cinnamon is a natural antimicrobial. A potent inhibitor of bacterial infection endotoxin is present in cinnamon bark. *Cinnamomum bejolghota *essential oils had activity against *Salmonella *spp. isolated from poultry (*S. agona*, *S. braenderup*, *S. derby*, *S. gallinarum*, *S. hadar*, *S. mbandaka*, *S. monterideo*, *S. saintpaul*, *S. schwarzergrund*, *S. senftenberg*) and *E. coli *O157. Pasteurized apple juice with nisin (0, 25, 50, 100, and 200 ppm, wt/vol) and cinnamon (0 and 0.3%, wt/vol) accelerates the death of *Salmonella typhimurium *and *E. coli *O157:H7 in apple juice enhancing product safety. Essential oils obtained from fresh leaves of *Cinnamomum aromaticum *were effective against the flagelated poultry parasites *Tetratrichomonas gallinarum *and *Histomonas meleagridis*. High level validity for diarrhea.	37-41
*Curcuma longa*	Curcumin, a yellow pigment of turmeric (*Curcuma longa*), is known to possess chemopreventive properties in various animal tumor models. Curcumin can effectively suppress the DEN-induced development of AHF in rat liver. The aqueous and alcoholic extracts isolated from turmeric are as effective as butylated hydroxy anisole in their anti-oxidative activity. There is a strong correlation between antioxidant activity and antiinflammatory activities of curcuminoids. Curcumin was also found to possess antiviral potential. *Curcuma longa *has an anti-thrombotic effect in mice. Mid level validity for proud flesh and caprine arthritis and as a palliative.	42-46
*Cymbopogon nardus*	Mosquito coils made from the leaves of *Cymbopogon nardus *had moderate knockingdown but insignificant killing effects on *Aedes aegypti*. High concentrations of *C. nardus*, were effective when screened against the mosquito *Aedes aegypti *under laboratory conditions using human subjects. *Cymbopogon nardus *provided at least 2 h complete repellency. The protection times of this oil was less when diluted. At 50% concentration, *C. nardus *showed 50 min protection and the repellent activity decreased to 30 min or less when diluted to 10%. The undiluted oil of *C. nardus *provided better protection against *Ae. aegypti, Cx quinquefasciatus *and *An. dirus*. Mid level validity as a fly repellent.	47, 48
*Cytisus scoparius*	Broom (*Cytisus scoparius*, syn. *Sarothamnus scoparius*) is a leguminous species with low contents of extractable condensed tannins, which would be unlikely to affect the digestion of nutrients in ruminants and has a high protein content. The young shoot tips of the broom, *Cytisus scoparius*, contain greater concentrations of sparteine than older leaves. Sparteine and the analogue BRB-I-28 produced a dose-dependent reduction in heart rate and blood pressure over the dose range 1–64 mumol/kg/min in pentobarbitone-anaesthetized rats subjected to left-ventricle electrical stimulation and occlusion of the left anterior descending coronary artery. High level validity as a cardiac tonic and browse plant.	49-51
*Daucus carota*	The LC50 values for *Daucus carota *against 4th instars of *Culex annulirostris *using acetone, ethanol, hexane, and methanol extracts were 236.00, 36.59, 77.19, and 241.8 mg/liter, respectively. Mid level validity for endoparasites.	52
*Echinacea purpurea*	*Echinacea purpurea *has been investigated for its potential to enhance immune function, primarily through activation of innate immune responses. A time course study, using the time of SRBC immunization to mimic the onset of illness, examined the effects of 8 and 4 days of *Echinacea purpurea *treatment at 0.6 mL/kg/day. Only in the 4-day administration, with dosing beginning 1 hour after SRBC immunization, was there an observed enhancement of the antibody forming cell response. This supports the acute use of *Echinacea purpurea *as suggested by anecdotal reports, and demonstrates the potential for enhancement of humoral immune responses as well as innate immune responses. High level validity for immune protection.	53
*Epilobium angustifolium*	Fireweed (*Epilobium angustifolium*) has an abundance of vitamin C and was used by Indians and settlers who picked and boiled the fresh green sprouts in springtime. A tea was also made from the leaves. Fireweed (*Epilobium angustifolium *L.) extracts showed inhibitory activity against metallopeptidases. Fireweed contains several flavonoids and phenolic acids and an ellagitannin with anti-inflammatory activity. The dimeric macrocyclic ellagitannin oenothein B and other polyphenols may partly support the use of *Epilobium *extracts in folk medicine for prostate conditions. *Epilobium angustifolium*, *Epilobium hirsutum*, *Epilobium palustre*, *Epilobium tetragonum *and *Epilobium rosmarinifolium *ethanolic extracts showed antimicrobial activity in a range of concentrations between 10 and 650 microgml of dry extract. *Epilobium angustifolium *and *Epilobium rosmarinifolium *had broad spectrum activity against bacteria, yeasts and fungi. The analgesic properties of *Epilobium angustifolium *(Ea) was established using the dry extract of Ea obtained by evaporating a commercially available mother tincture. High level validity as a tonic feed.	54-56
*Equisetum arvense*	The short-term actions of *Equisetum arvense *and *Lavandula officinalis *dry extracts, and of isoquercitrin, a flavonoid found in *Equisetum arvense*, on *in vitro *fermentation by rumen microbes was investigated. The addition of *Lavandula officinalis *and *Equisetum arvense *enhanced the fermentation rate of the hay only substrate by 50%, through an increased release of acetate and propionate. Isoquercitrin lowered the fermentation rate of the other two diets. High level validity as a source of minerals.	57
*Eugenia *caryophyllata (synonym *Syzygium aromaticum *L., *Eugenia aromatica *L., *Caryophyllus aromaticus *L.)	The antibacterial activity of different extracts of *Eugenia caryophyllata *was demonstrated against pathogenic bacteria. The fungicidal activity of the essential oil of *E. caryophyllata *was demonstrated against several food-borne fungal species, on fungi isolated from onychomicosis and on the yeast model *Saccharomyces cerevisiae*. The inhibition of adult emergence by *E. caryophyllata *extracts was demonstrated on *Culex pipiens *larvae. The essential oil of this plant showed repellency on the mosquitoes *Ades aegypti*, *Culex quinquefasciatus *and *Anopheles dirus*. Extracts showed insecticidal activity on *Pediculus capitis *and acaricidal activity on *Dermatophagoides farinae *and *D. pteronyssinus*. High level validity as a fly repellent.	58-64
*Euphrasia officinalis*	The major bioactive components in *Euphrasia *species are tannins, phenolic acids, flavones and iridoid glycosides. Compounds show a variety of effects including anti-inflammation, antioxidant, antibacterial, antiallergic, asthma and antihistamine activity. Eye drops made from *Euphrasia rostkoviana *Hayne have been used in anthroposophical medicine for more than 70 years for the structuring of the fluid organism in the eye, especially in inflammatory and catarrhal conjunctivitis. A prospective cohort trial was undertaken to describe the efficacy and tolerability of these eye drops in a community-based setting. Sixty-five (65) patients were involved. Complete recovery was seen in 53 patients (81.5%) and a clear improvement in 11 patients (17.0%). No serious adverse events were observed. A dosage of one drop three times a day was the general prescribed dosage. High level validity for eye problems.	65, 66
*Fucus *sp.	*Fucus vesiculosus *has antioxidant activity. High level validity as a feed supplement.	67
*Galium aparine*	Water distilled essential oils from aerial parts of *Galium aparine *and *Galium odoratum *contained seventy-two compounds. The major component of the essential oil of *G. aparine *was hexadecanoic acid (22.3%), and the major components of the essential oil of *G. odoratum *were thymol (30.6%) and isothymol (22.8%). *Galium aparine *oil contained mostly fatty acids and four terpenoids. The major components of the oil of *G. odoratuni *were thymol (30.6%) and isothymol (22.8%). Low level validity for mastitis.	70
*Gaultheria shallon*	High antioxidant activity was obtained from the extracts of *Gaultheria shallon*. Catechin and epicatechin, potent polyphenolic antioxidants, were identified in the EtOAc extracts of *Gaultheria shallon*. *Gaultheria shallon *fruits have high antioxidant activity and vitamin C. Salal foliage contains 21% condensed tannins by weight. High level validity as a feed supplement.	68, 69
*Hedera helix*	The secretolytic and bronchodilating properties found in *Hedera helix *extract are due to isaponins, especially alfa hederin. *H. helix *decreased arterial pressure in cats and also decreased stomach ulcer formation in rats. Extracts from *H. helix *wood presented spasmolytic, anti-inflammatory and anti-tussive activity. The sapogenins of *Hedera helix *L., non-competitively inhibit hyaluronidase activity in a dose-dependent fashion, showing comparable IC50 values (hederagenin IC50 = 280.4 microM; oleanolic acid IC50 = 300.2 microM); the saponins hederacoside C and alpha-hederin are very weak inhibitors. Hyaluronidase, a proteoglycan-degrading enzyme, may have an influence on collagenolysis in bovine placenta and take part in the separation processes of the placenta in cows. High level validity for retained placenta.	71-73
*Helianthus annuus*	Salicylic acid (SA)-treated sunflower leaves displayed potent antimicrobial activity against a set of phytopathogens which was due to proteins of approximately 60 kDa. Seeds of *Helianthus *species contain trypsin and subtilisin which are used in plant defense. Sunflower has allelopathic compounds which may include phenols and terpenes. Mid level validity for endoparasites.	74-76
*Hypericum perforatum*	The flowering tops of *Hypericum perforatum *contain a resinous substance, hypericine and pseudohypericine, a flavonoid, hyperoxide, essential oil, tannic and mucilaginous substances. The resin and the essence contribute to the vulnerary and epithelising properties of the plant and explain its use in folk phytotherapy as a topical remedy against ulceration and burns. An experiment was carried out on 24 female patients of a mean age of 33 ± 3 years, who had had a caesarean section. The tested substance was a mixture of 70% oily extract of Hypericum and 30% oily extract of *Calendula*. The surface perimeter area of the surgical wound in the group treated with the *Hypericum*-*Calendula *mixture was reduced by 37.6 ± 9.9% compared to a reduction of 15.83 ± 4.64% in the control group (wheat germ oil). High level validity for proud flesh and wounds.	77
*Juniperus communis*	Acetone extracts of the fruits of *Juniperus sabina *showed prominent antifeedant and stomach toxic effects to *Pieris rapae*. The extract also showed strong antifeedant activity against *Mythimna separata *Walker and *Plutella xylostella *L, inhibited the population growth of *Sitophilus zeamais *Motschulsky and *Tribolium castaneum *Herbst and disrupted the growth of *Helicoverpa armigera *Hübner. The insecticidal compound was identified as deoxypodophyllotoxin. Hexane and methanol extracts from *Juniperus communis *inhibited the growth of *Mycobacterium tuberculosis*. *Mycobacterium avium *was inhibited by *Juniperus communis *hexane extract. High level validity for endoparasites.	78, 79
*Lavandula officinalis*	*Lavandula *was used as a strewing herb due to its insect-repellent properties. The essential oil of *Lavandula officinalis *showed repellent activities against *Culex pipiens pallens *on hairless mice. Essential oils were extracted by steam distillation from flowers of *Lavandula stoechas*. Compounds found were fenchone, 1,8-cineole, bornyl acetate, myrtenyl acetate, myrtenol, alpha-pinene and viridiflorol. High level validity as a fly repellent. Mid level validity for proud flesh.	12, 80, 81
*Malva*sp.	Dioscorides, Pliny and Arab physicians described similar medicinal uses for *Malva *as the ethnoveterinary uses in this paper. Hexane extracts from *Malva parviflora *inhibited the growth of *Mycobacterium tuberculosis*. *Mycobacterium avium *was inhibited by the methanol extract of *Malva parviflora*. Aerial parts of *Malva neglecta *protected two of six rat stomachs from ethanol-induced ulcerogenesis. Hexane and methanol extracts made from the roots of *Malva parviflora *were active against both Gram-positive and Gram-negative bacteria. These extracts also had high cox-1 inhibiting activity. Extracts made from the creeping prostate and upright forms showed variation in antibacterial activity but the cox-1 anti-inflammatory activity was similar for all of the extracts. High level validity for wounds.	9, 82,83, 79
*Matricaria chamomilla*	A comprehensive review of chamomile was published in 2006. Chamomile flowers contain more than 120 constituents. The flower head contains 10% mucilage, which in turn consists of amino acids, polysaccharides and fatty acids. The compounds found in the essential oil derived from the flowers include the terpenoids alpha-bisabolol and its oxides and azulenes, including matricin. The antioxidant capacity of chamomile is relatively low (<18 mmol/100 g). German chamomile oils (*Matricaria chamomilla*) were slightly more effective against 25 different Gram-positive and Gram-negative bacteria and 20 strains of *Listeria monocytogenes *than oil from Roman 'chamomile' (*Chamaemelum nobile*). Chamomile aqueous extracts showed significant antiplatelet activity *in vitro*. A freeze-dried extract of chamomile given to Wistar albino rats suppressed both the inflammatory effect and leukocyte infiltration induced by a simultaneous injection of carrageenan and prostaglandin E1. Mid level validity for eye problems.	84
*Medicago sativa*	Cattle fed diets high in Se from agricultural products such as high Se wheat and alfalfa hay will accumulate substantial amounts of Se in the meat without developing signs of Se toxicity. High level validity for selenium deficiency.	85
*Melaleuca alternifolia*	*Melaleuca alternifoli*a Cheel essential oil and its major component terpinen-4-ol had anti-staphylococcal activity against strains resistant to mupirocin, fusidic acid, vancomycin, methicillin and linezolid. *Melaleuca alternifoli*a oil has antiprotozoal activity. *Melaleuca alternifoli*a oilcaused a 50% reduction in growth (compared to controls) of the protozoa *Leishmania major *and *Trypanosoma brucei *at concentrations of 403 mg/ml and 0.5 mg/ml, respectively. This activity was attributed to terpinen-4-ol. *Melaleuca alternifoli*a oil at 300 mg/ml killed all cells of *Trichomonas vaginalis*. High level validity for endoparasites.	86, 87
*Melissa officinalis*	Lemon balm tea reportedly gives long life by dispelling melancholy. *Melissa officinalis *(lemon balm) and *Valeriana officinalis *(valerian) were assessed on their anxiolytic properties during laboratory-induced stress in a double-blind, placebo-controlled, randomized, balanced cross-over experiment. 24 healthy volunteers received three separate single doses (600 mg, 1200 mg, 1800 mg) of a standardized product containing *M. officinalis *and *V. officinalis *extracts, plus a placebo, on separate days separated by a 7 day wash out period. The 600 mg dose of the combination product ameliorated the negative effects of the stress. The highest dose (1800 mg) produced an increase in anxiety. High level validity for anxiety.	12, 88
*Mentha piperita, Mentha pulegium*	Pulegium was named by Pliny for its reputation of driving away fleas. *Mentha piperita *is effective in controlling the larvae of *C. quinquefasciatus *Say. Extracts of *Mentha longifolia *(L.) Huds., *Melissa officinalis *L., and *Mentha pulegium *L. were tested against the house mosquito *C. pipiens*. Ethanol extracts of *Melissa officinalis*, *Mentha longifolia *exhibited complete (100%) larvicidal activity at 200 ppm. At this concentration, mortality was not significantly different from that of the reference temephos, although 200-fold more material was needed to achieve that result. At this same concentration *Mentha pulegium *extracts resulted in 90% mortality. In addition, the extracts of *Mentha longifolia *and *Melissa officinalis *also showed good (>85%) larvicidal activity at 100 ppm. The volatile oils of *Mentha microphylla *was tested against adult *Lucilia sericata *implicated in myiasis. The LC50 was 130 ppm by *Mentha microphylla*. High level validity as a fly repellent.	9, 89, 90
*Nepeta caesarea*	The Roman town of Nepeti grew catnip as a medicine. The leaves contain vitamin C and the infusion reportedly relieves colds by inducing sleep and increasing perspiration without a corresponding body temperature increase. *Nepeta caesarea *showed significant analgesic activity, besides marked sedation, which was also blocked by naloxone, indicating involvement of opioid receptors but excluding mu-opioid receptors. The main antinociceptive component of the plant is nepetalactone. High level validity for pain relief.	12, 91, 92
*Origanum *× *majoricum*	The medicinal properties of *Origanum *were known to the ancient Greeks and Egyptians. Sweet marjoram was introduced to Europe during the Middle Ages. *Origanum *× *majoricum*, *Origanum vulgare *ssp. *hirtum*, and *Poliomintha longiflora *have higher phenolic contents as compared to other culinary herbs. Rosmarinic acid was the predominant phenolic compound in *Salvia officinalis*, *Thymus vulgaris *and *Origanum *× *majoricum*. High level validity as a feed supplement.	12, 93
*Pastinaca sativa*	There are at least seven furanocoumarins present in green tissues of wild parsnip that deter plant pests. Mid level validity for endoparasites.	94
*Petroselinum crispum*	Homer reported that warriors fed parsley to their horses. *Petroselinum crispum *produces a complex mixture of phenylpropanoids, coumarins, and terpenoids. The tested species contained phenylpropanoids, myristicin and parsley apiole; three linear furanocoumarins, xanthotoxin, imperatorin, and bergapten and two monoterpenes. The myristicin from parsley oil showed insecticidal activity. Mid level validity for endoparasites.	12, 95
*Pinus ponderosa*	Pine oil had larvicidal activity against mosquitoes with LC50 values ranging between 82 and 112 ppm. The pine oil provided 100% repellent protection against *Anopheles culicifacies *for 11 h and 97% protection against *Culex quinquefasciatus *for nine hours. Pycnogenol^® ^is a phytochemical extracted from the bark of *Pinus pinaster *Ait. Pycnogenol^® ^consists of standardized proportions of monomeric and oligomeric procyanidins and phenolic acids (derivatives of benzoic acid and cinnamic acid). Pycnogenol was tested for its antimicrobial activity against 23 different pathogenic prokaryotic (gram-positive and gram-negative) and eukaryotic (yeast and fungi) microorganisms. Pycnogenol inhibited the growth of all the tested microorganisms in minimum concentrations ranging from 20 to 250 microg/mL. Dilution of the Pycnogenol^®^-containing media re-initiated the proliferation of microorganisms. High level validity for diarrhea.	96, 97
*Plantago major*	EH0202 is a health-food additive from Japan. It is a mixture of four herbal extracts known to stimulate macrophage activity (interferon inducers). They are: pumpkin seeds (*Cucurbita moschata*), plantain seeds (*Plantago asiatica*), Japanese honeysuckle (*Lonicera japonica*), and safflower (*Carthamus tinctorius*). EH0202 administration decreases the incidence of viral pneumonia and the mortality rate in pigs with porcine reproductive and respiratory syndrome. EH0202 acts to stimulate immunological systems and may improve endocrine dysfunction. Hot water extracts of *Plantago major *and *Plantago asiatica *were investigated *in vitro *on herpesviruses (HSV-1 and HSV-2) and adenoviruses (ADV-3, ADV-8 and ADV-11). The hot water extract of *Plantago asiatica *possessed significant inhibitory activity on viral infection (HSV-2 and ADV-11). *Plantago major *and *Plantago asiatica *both showed dual effects of immunodulatory activity, enhancing lymphocyte proliferation and secretion of interferon-gamma at low concentrations (< 50 microg/ml), but inhibiting this effect at high concentration (> 50 microg/ml). High level validity for diarrhea.	98, 99
*Polystichum munitum*	The acetone extract of *Polystichum pungens *inhibited five gram-positive bacteria. *Bacillus cereus*, *Bacillus pumilus*, *Bacillus subtilis*, *Micrococcus kristinae *and *Staphylococcus aureus*. The methanol extracts of *Polystichum pungens *inhibited the growth of both the gram-positive as well as the gram-negative bacteria, with the exception of *E. coli *at 5.0 mg/ml. The water extract of *Polystichum pungens *showed activity against four of the gram-positive bacteria and *Enterobacter cloacae*. *Polystichum squarrosum *is asssociated with microscopic enzootic bovine haematuria in cattle. Mid level validity as a digestive stimulant.	100, 101
*Portulaca oleracea*	*Portulaca oleracea *and *Portulaca intraterranea *have a zinc content of 6.5 mg/100 g. The genus *Portulaca *contains oxalates and an oxalic acid content of up to 9%. These plants also contain alkaloids, coumarins, flavonoids and anthraquinone glycosides. *Portulaca oleracea *nutritive values are: ash (32.5%), crude protein (17.9%), ether extract (5.6%), crude fibre (20.3%), moisture (97.3%) soluble carbohydrate (23.6%), calcium (1.8%), magnesium (3.5%), phosphorus (0.3%), and calcium: phosphorus ratio (5.9%). Nubian goats fed fresh *Portulaca oleracea *(5 g/kg BW) showed weakness of the fore and hind limbs with inability to stand, greenish watery diarrhoea and polyuria. The aqueous extract of the *Portulaca oleracea *leaves and stems might act in part on postsynaptic a-adrenoceptors and interfere with transmembrane calcium influx. The plant was not recommended for daily use when fresh and in large quantities. Mid level validity for zinc deficiency.	102, 103
*Potentilla tormentilla, Potentilla pacifica*	The name Tormentil is said to come from the Latin *tormentum*, referring to the gripings of the intestines that the herb will serve to relieve. A randomized, double blinded, placebo-controlled trial was conducted at Children's Hospital for Infectious Diseases #3, St. Petersburg, Russia in 40 children ranging in age from 3 months to 7 years with rotavirus diarrhea. There were 2 comparison groups: a treatment group that consisted of 20 children treated with tormentil root extract (*Potentilla tormentilla*); and a control group of 20 children who received a placebo. Administration of tormentil root extract in controlled doses shortened the duration of rotavirus diarrhea and decreased the requirement for rehydration solutions. Tormentil root extract was said to be an effective treatment for rotavirus diarrhea in children. A root extract of *Potentilla arguta *completely inhibited respiratory syncytial virus. High level validity as an appetite stimulant.	9, 104, 105
*Prunella vulgaris*	Gerard describes *Prunella *as a wound herb. *Prunella vulgaris *L. contains polysaccharides with antiviral activity. *Prunella vulgaris *ontains oleanolic, betulinic, ursolic, rosmarinic (antioxidant), caffeic and other acids, triterpenoids, flavonoids, tannins and the antiviral polysaccharide prunelline. The aqueous fraction of the plant inhibits anaphylactic shock, allergic reactions, protects rat erythrocytes against haemolysis and kidney and brain homogenates against lipid peroxidation. Antimicrobial activity was also found. This study concluded that the ethnomedicinal use of *Prunella vulgaris *for wound healing and as an anti-inflammatory remedy is supported. High level validity for wounds.	9,106, 107
*Pseudotsuga menziesii*	*Pseudotsuga menziesii *oils contain about 60 compounds with monoterpenes (especially sabinene and beta-pinene) as the major constituents. These had antimicrobial effects against bacteria, fungi and worms. Mid level validity for coccidiosis, endoparasites and as an appetite stimulant.	108
*Quercus alba*	*Quercus robur *leaves contain 141 ± 16 ppm (dry weight) zinc. Mid level validity for zinc deficiency.	109
*Rosa nutkana*	The extracts of *Rosa nutkana *and *Amelanchier alnifolia *were very active against an enteric coronavirus. High level validity as a feed supplement.	105
*Rubus ursinus*, *Rubus laciniatus*	Pharmacological studies of the leaf extract of *Rubus idaeus *on the uterus *in vitro *and other smooth muscle preparations have found activity. Specific compounds in *Rubus pinfaensis *(triterpenoids, phenols) and *Rubus imperialis *(triterpenes) have antibacterial and antinociceptive properties, respectively. The leaves of *Rubus idaeus *have volatile compounds and waxes. Diterpene glycosides are found in the leaves of *Rubus chingii *and *Rubus suavissimus *and triterpenes in the leaves of *Rubus imperialis *and *Rubus pinfaensis*. Compounds in the leaves of *Rubus idaeus*, produce a relaxant response on a transmurally stimulated guinea-pig ileum *in vitro*, and are polar in nature. Mid level validity for milk production and to treat unknown illnesses.	110
*Ruta graveolens*	Rue has historically been a strewing herb and anti-plague plant. Common rue (*Ruta graveolens*) has an antifeedant activity against mahogany shootborer larvae (*Hypsipyla grandella*). Mid level validity as a fly repellent.	12, 111
*Salix *sp.	The principal active component of *Salix *sp. is salicin, however the species also contains phenolic glycosides (salicortin, fragilin, tremulacin) in the bark. A standardized willow bark extract was examined in 127 outpatients with osteoarthritis and rheumatoid arthritis in 2 randomized, controlled, double-blind trials with follow up for 6 weeks. The difference between willow bark extract and placebo was not statistically significant in either trial. Ethanolic *Salix *extract 1520L inhibits COX-2-mediated PGE2 release through compounds other than salicin or salicylate. The *Salix *extract is a weak inhibitor of pro-inflammatory cytokines. Mid level validity for mastitis and unknown illnesses.	92, 112-114
*Salix *sp.	210 patients with an exacerbation of chronic low back pain who reported current pain of 5 or more (out of 10) on a visual analog scale were randomly assigned to receive an oral willow bark extract with either 120 mg (low dose) or 240 mg (high dose) of salicin, or placebo, with tramadol as the sole rescue medication, in a 4-week blinded trial. The principal outcome measure was the proportion of patients who were pain-free without tramadol for at least 5 days during the final week of the study. The numbers of pain-free patients in the last week of treatment were 27 (39%) of 65 in the group receiving high-dose extract, 15 (21%) of 67 in the group receiving low-dose extract, and 4 (6%) of 59 in the placebo group (P < 0.001). Significantly more patients in the placebo group required tramadol (P < 0.001) during each week of the study. One patient suffered a severe allergic reaction, perhaps to the extract. High level validity for pain. Mid level validity for caprine arthritis.	114
*Salvia *sp.	In the 17^th ^century the Dutch found that the Chinese would trade three chests of tea for one of sage leaves. The ingestion of 200, 400 and 800 mg/kg of aqueous or 400 mg/kg of ethanolic extracts of *Salvia fruticosa *from day one to day six of pregnancy by female rats did not cause pregnancy failure. However, the ingestion of an ethanolic extract reduced the number of viable fetuses and increased the number of resorptions in the pregnant rats. A highly significant fetal resorptive effect was seen with the ethanolic extract, with 37% fetuses degenerated, while the aqueous extract showed significant activity with 31% of fetuses resorbed. The ingestion of *Salvia fruticosa *by adult male rats had no effect on the fertility of females impregnated by the treated males. However, the number of implantation sites and the number of viable fetuses were reduced. These losses appear to be due to either faulty preimplantation development or decrease in sperm function. Mid level validity for drying off.	12, 115
*Senna *sp.	Ten Nubian goats were given oral doses of the fresh fruits and leaves of *Cassia senna *at 1, 5, and 10 g/kg/day. Eight goats died within 30 days and two others were slaughtered in a poor condition on days 18 and 29. The clinical signs shown were diarrhoea, inappetence, loss of condition, and dyspnea. *Senna *is not carcinogenic to rats given dosages of up to 300 mg/kg/day daily for 2 years. Mid level validity for diarrhea.	116, 117
*Symphoricarposalbus *var. *laevigatus*	*Symphoricarpos albus *was found to have phenolic acids in the extracts and fractions from leaves, flowers and fruit with antimicrobial activity. Mid level validity for endoparasites.	119
*Symphytum officinale*	The ethnoveterinary uses of comfrey are related to medicinal uses recorded in Gerard and Culpepper. The antiinflammatory activity of comfrey (*Symphytum officinale*) is linked to rosmarinic acid, which has antioxidant, antiviral, bactericidal and viricidal activities. The soothing and wound healing properties are due to allantoin with reported anti-inflammatory, immunostimulant and vulnerary activities. Mid level validity as a laxative, for ketosis and to increase butterfat. High level validity for proud flesh, wounds and udder edema.	9, 120
*Syzygium aromaticum*	Undiluted clove oil gave the longest duration of 100% repellency (2–4 h) against all tested species of mosquito: *Aedes aegypti*, *Culex quinquefasciatus *and *Anopheles dirus*. Low level validity for coccidia.	48
*Taraxacum officinale*	Dandelion was first described as a medicine by Arabian physicians of the tenth and eleventh centuries. A comprehensive review of all studies conducted on dandelion has been recently published. One study found a partial inhibition of rat paw oedema induced by carrageenan and following intraperitoneal treatment with 100 mg/kg dm. A dried 80% ethanolic extract of *Taraxacum officinale *root administered orally at 100 mg/kg body weight 1 h before oedema elicitation inhibited carrageena ninduced rat paw oedema by 25%, versus a 45% inhibition with indomethacin at 5 mg/kg. The methanolic extract of flowers of *Taraxacum officinale *and *Taraxacum platycarpum *showed inhibition rates of 95 and 87%, respectively, of tetradecanoylphorbol-13-acetate (TPA)-induced ear oedema in mice. The triterpene uvaol isolated from dried flowers of *Taraxacum platycarpum *inhibited the TPA-induced inflammation at an equivalent level to indomethacin with 0.1 mg/ear being the 50% inhibitory dose. Extracts of *Taraxacum officinale *leaf and roots exhibited slightly lower inhibition rates of 69 and 51%, respectively, in the same assay. Dandelion leaf extract was also shown to have an anti-inflammatory activity on the central nervous system. Mid level validity for udder edema and high level validity as a feed.	9, 121
*Teucrium scorodonia*	Ethyl acetate, chloroform and n-butanol extracts of *Teucrium montanum *showed a wide range of inhibiting activity against both Gram (+) and Gram (-) bacteria. Mid level validity for mastitis.	122
*Thuja plicata*	Ethanolic and acetone extracts of *Thuja orientelis *were studied against III instar larvae of *Anopheles stephensi *and *Culex quinquefasciatus*. The ethanolic extract of T. orientelis was effective against both larval species with LC50 values of 13.10 and 9.02 ppm after 24 and 48 hours for anopheline and 22.74 and 16.72 ppm against culicine larvae. The acetone extract showed LC50 values of 200.87 and 127.53 ppm against anopheline and 69.03 and 51.14 ppm against culicine larvae. Mid level validity for endoparasites and lice. Low level validity for copper deficiency.	123
*Ulmus fulva*	*Ulmus macrocarpa *Hance has low to moderate anti-protozoal efficacy against *Toxoplasma gondii *and *Neospora caninum*. One-day-old broiler chicks were infected with *Eimeria tenella *and given various herbal extracts. Survival rates, lesion scores, body weight gains, bloody diarrhea, and oocysts excretions were investigated at the first and the second week after infection. All the birds treated with *Ulmus macrocarpa *survived. Lesion scores in the groups treated with *Ulmus macrocarpa *(1.40 +/- 1.14) were lower than the control. Mid level validity for endoparasites.	124, 125
*Urtica dioica*	*Urtica dioica *is reported to have anti-inflammatory, acute diuretic, natriuretic and hypotensive effects. The phenolic compounds present in *Urtica dioica *L. may contribute to its antioxidant activity. A water extract of *Urtica dioica *showed antimicrobial activity against 9 Gram-positive and Gram-negative bacteria and one yeast. A water extract of *Urtica dioica *also showed antiulcer activity against ethanol-induced ulcerogenesis and an analgesic effect. *Ultica dioica *agglutinin, a plant lectin, consists of seven individual isolectins. Isolectin I binds Zn(2+) ions. Mid level validity for zinc deficiency. Mid level validity for immune system protection. High level validity as a tonic and for diarrhea. Low level validity for endoparasites.	126, 127
*Urtica dioica*	A multicenter, prospective clinical trial was performed on 257 patients to study the efficacy and tolerance of a compound drug PRO 160/120 (*Sabal palmetto *and nettle) in elderly men with lower urinary tract symptoms due to benign prostatic hyperplasia. Group 1 of 129 patients received PRO 160/120 which was found to be superior to the placebo. High level validity as a tonic and for diarrhea.	128
*Usnea longissima*, *Usnea barbata*	*Usnea barbata *(L.) Mott and *Usnea hirta *(L.) Wigg hydroalcoholic extracts have antiinflammatory activity comparable to phenylbutazone and hydrocortisone hemisuccinate; the analgesic activity was close to that of noraminophenazone; the antipyretic activity was equivalent or better than aminophenazone. *Usnea hirta *has usnic, thamnolic and usnaric acids with antibiotic effects. *Usnea longissima *contains usnic and evernic acids which act as expectorants. Usnic acid has 2 enantiomeric forms with different activities including antimicrobial activity against Gram-positive and anaerobic bacteria including antibiotic-resistant pathogenic strains. It also has antiviral, antiprotozoal, antiproliferative, anti-inflammatory (equivalent to ibuprofen) and analgesic activity. High level validity for wounds and foot rot.	129-131
*Vaccinium *sp.	The compounds absorbed into the rat blood after oral administration of ethanol extract of the stems and leaves of *Vaccinium vitis-idaea *were analyzed. Two compounds found in the plasma were arbutin and fraxin. Both arbutin and fraxin have anti-inflammatory, anti-coughing and phlegm-removing effects. Fraxin at the higher dosage tested had similar activity to dexamethasone; arbutin was less active. Docosane, quercetin, daucosterol, hyperoside, have also been isolated from the stem and leaf of the plant. Two huckleberry species, *Vaccinium membranaceum *and *Vaccinium ovatum *were evaluated for their total, and individual, anthocyanin and polyphenolic compositions. *Vaccinium ovatum *had greater total anthocyanin, total phenolics, oxygen radical absorbing capacity, and ferric reducing antioxidant potential than *Vaccinium membranaceum*. The pH and degrees Brix were also higher in *Vaccinium ovatum*. Each species contained 15 anthocyanins (galactoside, glucoside, and arabinoside of delphinidin, cyanidin, petunidin, peonidin, and malvidin) but in different quantities. They also had a different polyphenolic profile. The polyphenolics of both species had a high proportion of cinnamic acid derivatives and flavonol glycosides. The major polyphenolic compound in V. membranaceum was neochlorogenic acid, and in *Vaccinium ovatum*, chlorogenic acid. Mid level validity for ketosis and as a feed supplement.	132, 133
*Valeriana officinalis*	Valerian was used in World Wars 1 and II to treat shell shock. A review of *Valeriana officinalis *states that the compounds in the volatile oil vary due to genetics and environmental causes. Main constituents include the monoterpene bornyl acetate and the sesquiterpene valerenic acid. Some sesquiterpenes act on the amygdaloid body of the brain and valerenic acid inhibits enzyme-induced breakdown of GABA in the brain producing sedation. The valepotriates are changed into homobaldrinal which reduces the spontaneous motility of mice. The aqueous extracts of the roots contain GABA which could cause sedation depending on its bioavailability. A lignan, hydroxypinoresinol, can bind to benzodiazepine receptors.	12, 134
*Verbascum thapsus*	The use of mullein for respiratory problems is derived from traditional folk medicine. Extracts of *Verbascum thapsus *exhibited antiviral activity against herpesvirus type 1 and influenza viruses. High level validity as a respiratory tonic.	12, 135
*Vitis *sp.	The components of the pure plant-based extract AS 195 (Folia vitis viniferae) are flavon(ol)-glycosides and glucuronides with quercetin-3-O-beta-D-glucuronide (main flavonoid) and isoquercitrin (quercetin-3-O-beta-glycoside; a secondary flavonoid). Low level validity for unknown illnesses.	136
*Zea mays*	The use of the stigma and styles of *Zea mays *as a diuretic is found only in those parts of Italy where the Spanish influence was strong. This ethnomedicinal use is also found in the Caribbean and in Latin America and is still found in Spain. Corn silk aqueous extract is diuretic in rats at large dosages. Mid level validity for udder edema.	137, 138
*Zingiber officinale*	*Zingiber officinale *is active against *Helicobacter pylori *strains, and also has antiinflammatory, antioxidant and antitumoral activity. An extract from the root of *Zingiber officinale *reduced the minimum inhibitory concentrations of aminoglycosides in vancomycin-resistant enterococci. The effective compound [10]-gingerol with its detergent-like effect potentiated the antimicrobial activity of the aminoglycosides. High level validity for diarrhea and scours.	139, 140

We suspected that traditional medicines in British Columbia are derived from the knowledge and traditions of First Nations peoples, and from Asia and Europe. Elders of the Saanich and Cowichan Coast Salish people of southern Vancouver Island treat, or have treated in the recent past, many ailments with bark preparations [6,10,11]. Respiratory ailments were treated with bark of *Abies grandis*, *Arbutus menziesii*, *Cornus nuttallii*, *Prunus emarginata*, *Pseudotsuga menziesii *and *Quercus garryana*, digestive tract ailments with the bark of *Abies grandis*, *Alnus rubra*, *Arbutus menziesii*, *Malus fusca*, *Oemleria cerasiformis*, *Populus tremuloides*, *Pseudotsuga menziesii*, *Rhamnus purshianus *and *Rubus spectabilis*, gynaecological problems with bark of *Abies grandis*, *Arbutus menziesii*, *Populus tremuloides*, *Prunus emarginata*, *Pseudotsuga menziesii *and *Sambucus racemosa*, and dermatological complaints with the bark of *Mahonia *spp., *Rubus spectabilis*, and *Symphoricarpos albus*.

One First Nation group used medicinal preparations from *Arbutus menziesii *bark and leaves for colds, stomach problems, as a post-childbirth contraceptive, and in a ten-ingredient bark medicine for tuberculosis and spitting up blood [6,10,11]. Tree barks have also been used to treat fevers, diabetes, kidney problems, sore eyes, and haemorrhaging, and also as general tonics. In most cases, infusions or decoctions of barks are used. The medicines are drunk or applied externally as a wash. Several of these uses are similar to the ethnoveterinary uses described in this paper. These commonalities and those with European folk medicine will be discussed in more detail in future publications.

## Conclusion

This research was undertaken with the understanding that the use of safe and effective medicinal plants can reduce farmers' input costs, preserve the resource base, enhance biodiversity and protect animal health. If plants are grown on-farm this will enhance the biological interactions on which productive agriculture depends. Successful medicinal plant use can contribute to farm incomes, maintain the resilience of farm communities, promote self-reliance and contribute to an internationally recognized safe and good quality food supply, in addition to providing improved and affordable livestock health care. It can also strengthen rural community capacity building, leadership and skills development and help preserve the ethnomedicinal heritage of British Columbia.

Ethnoveterinary alternatives (based on medicinal plants) are an option for small-scale livestock farmers who cannot use allopathic drugs or for those larger conventional farmers whose economic circumstances prevent the use of veterinary services for minor health problems of livestock. Participatory workshops in combination with non-experimental validation are an effective means of producing information to be disseminated to farmers in a user-friendly format. Scientists may be motivated to conduct formal validation on plants that they know are being used for specific purposes.

The majority of the plants were used for goats. This reflects the browsing nature of the goat and the corresponding need for their owners to monitor what they were browsing and its constituents. Goats and sheep were the main species medicated or self-medicated on the Pinaceae, Cupressaceae and Ericaceae.

The majority of the plants achieved the mid to high levels of validity. This may be due to the fact that the majority of the respondents were referring to published material [5, 6, 7 and 120 among others] in their decision making. Some of the plants showing high levels of validity were *Hedera helix *for retained placenta and *Euphrasia officinalis *for eye problems. Plants with high validity for wounds and injuries included *Hypericum perforatum*, *Symphytum officinale*, *Usnea *spp., *Malva parviflora *and *Prunella vulgaris*. Treatments with high validity against endoparasites included those with *Juniperus communis *and *Pinus ponderosa*. Anxiety and pain are well treated with *Valeriana officinalis*, *Melissa officinalis *and *Nepeta caesarea. Verbascum thapsus *has high level validity as a respiratory tonic.*Zingiber officinale *is a good, but possibly expensive, treatment for diarrhea as are the other spices used. This high level of correspondence with the published literature is a reflection of the many ancient folk traditional practices that have been translated into ethnoveterinary practices and also reflects the recent scientific interest in subjecting medicinal plants to clinical trials.

In the participatory manual that we produced from this research and gave to participants, we cautioned against giving goats large amounts of red cedar (*Thuja plicata*) in early pregnancy (first six weeks) because of a neurotoxin in the plant. Red cedar (*Thuja plicata*) gives the milk of dairy animals a pitchy flavour. Respondents were initially concerned about the safety of Western hemlock (*Tsuga heterophylla*) branches fed to goats during pregnancy for its vitamin C content. Western yew foliage is poisonous to cattle and horses, the berries are poisonous.

Many plants designated as weeds by professionals (who have devoted considerable resources to understanding and eradicating them) are included in the diets of ruminants and the non-experimental validation of them suggests that they are nutritious and valuable feed supplements. The preliminary evaluation of the plants used for ruminants in British Columbia indicates that they are practical and possibly efficacious remedies that merit more formal evaluation.

## Competing interests

The author(s) declare that they have no competing interests.
